# {*N*,*N*′-[2,2′-(Ethane-1,2-diyldisulfanediyl)di-*o*-phenyl­ene]bis­(quinoline-2-carboxamidato)}copper(II)

**DOI:** 10.1107/S1600536811019581

**Published:** 2011-05-28

**Authors:** Soraia Meghdadi, Valiollah Mirkhani, Peter C. Ford

**Affiliations:** a Department of Chemistry, University of Isfahan, Isfahan 81746-73441, Iran; b Department of Chemistry and Biochemistry, University of California, Santa Barbara, California, 93106, USA

## Abstract

In the title compound, [Cu(C_34_H_24_N_4_O_2_S_2_)] or [Cu(bqdapte)], where H_2_bqdapte is 1,2-{bis­[2-(quinoline-2-carboxamido)­phen­yl]sulfan­yl}ethane, the Cu^II^ ion is coordinated to the dianionic hexa­dentate bqdapte^2−^ ligand by two amide and two quinoline N atoms and two thio­ether S atoms. In the observed conformation of the hexa­dentate ligand, the quinoline rings attain positions related by a twofold axis. The Cu atom displays a Jahn–Teller-distorted octa­hedral CuN_4_S_2_ geometry axially compressed along the two *trans*-configured Cu—N_amidate_ bonds.

## Related literature

For general background to the applications of transition metal complexes of hybrid *N*,*S*-donor ligands, see: Kouroulis *et al.* (2009 ▶); Lee *et al.* (2007 ▶); Ronson *et al.* (2006 ▶); Sarkar *et al.* (2009 ▶); Tavacoli *et al.* (2003 ▶); Xie *et al.* (2005 ▶). For related structures, see: Kouroulis *et al.* (2009 ▶); Sarkar *et al.* (2009 ▶); Singh & Mukherjee (2005 ▶); Sunatsuki *et al.* (1998 ▶); Zhang *et al.* (2004 ▶). For the synthesis of the ligand see: Meghdadi *et al.* (2011 ▶).
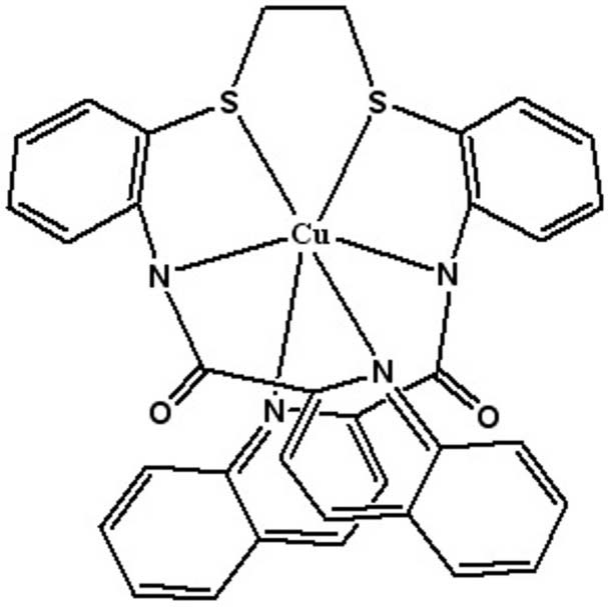


## Experimental

### 

#### Crystal data

[Cu(C_34_H_24_N_4_O_2_S_2_)]
*M* *_r_* = 648.23
Orthorhombic, 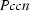

*a* = 11.4124 (15) Å
*b* = 13.5097 (18) Å
*c* = 18.606 (2) Å
*V* = 2868.6 (7) Å^3^
*Z* = 4
Mo *K*α radiation
μ = 0.95 mm^−1^
*T* = 150 K
0.30 × 0.25 × 0.08 mm

#### Data collection

Bruker SMART 100 diffractometer
Absorption correction: multi-scan (*SADABS*; Bruker, 2003 ▶) *T*_min_ = 0.770, *T*_max_ = 0.927
21464 measured reflections
2926 independent reflections
2467 reflections with *I* > 2σ(*I*)
*R*_int_ = 0.040

#### Refinement

*R*[*F* ^2^ > 2σ(*F* ^2^)] = 0.041
*wR*(*F* ^2^) = 0.119
*S* = 1.22
2926 reflections
195 parameters
H-atom parameters constrained
Δρ_max_ = 0.83 e Å^−3^
Δρ_min_ = −0.40 e Å^−3^

### 

Data collection: *SMART* (Bruker, 2003 ▶); cell refinement: *SAINT* (Bruker, 2003 ▶); data reduction: *SAINT*; program(s) used to solve structure: *SHELXS97* (Sheldrick, 2008 ▶); program(s) used to refine structure: *SHELXL97* (Sheldrick, 2008 ▶); molecular graphics: *SHELXTL* (Sheldrick, 2008 ▶); software used to prepare material for publication: *SHELXL97*.

## Supplementary Material

Crystal structure: contains datablocks I, global. DOI: 10.1107/S1600536811019581/qk2010sup1.cif

Structure factors: contains datablocks I. DOI: 10.1107/S1600536811019581/qk2010Isup2.hkl

Additional supplementary materials:  crystallographic information; 3D view; checkCIF report

## Figures and Tables

**Fig. 1.**
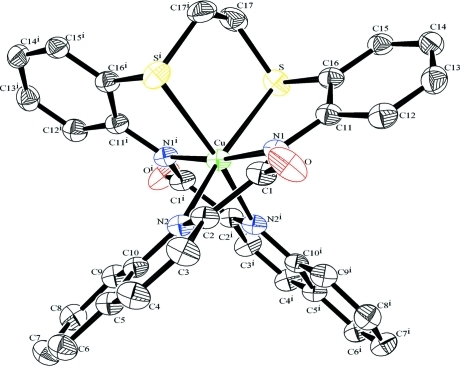
The ORTEP drawing of (I), with atom labeling scheme. Displacement ellipsoids are drawn at 50% probability level.

## supplementary crystallographic information

### Comment

The coordination chemistry of transition metal complexes with flexible hybrid N,*S*-donor ligands has been the focus of growing attention (Sarkar *et al*. 2009) due to their application in designing new molecular architectures (Ronson *et al.*, 2006; Tavacoli *et al.*., 2003; Xie *et al.*., 2005) and in bioinorganic chemistry (Kouroulis *et al.* 2009; Lee *et al.*, 2007). Many of these efforts have been devoted to the design and synthesis of new caboxamide ligands (Kouroulis *et al.* 2009; Singh & Mukherjee 2005; Sunatsuki *et al.*, 1998; Zhang *et al.*, 2004). The bioinorganic relevance of copper and its crucial role in many biological and catalytic functions have stimulated efforts towards the design, synthesis, and characterization of copper complexes as models for providing better understanding of biological systems and for the development of efficient catalysts (Lee *et al.*, 2007; Zhang *et al.*, 2004). In continuation of our studies on carboxamido metal complexes, we herein report the synthesis and structure of the title compound, [Cu(C_34_H_24_N_4_O_2_S_2_)], (I), and make a brief comparison with reported structures.

The structure of complex (I), and the atomic numbering used, is shown in Fig. 1. The Cu(II) ion displays a Jahn-Teller distorted octahedral CuN_4_S_2_ geometry arising from the hexadentate thiocarboxamido ligand. This complex has a 2-fold axis passing through Cu and the midpoint of C17 and its symmetry related atom. Two quinoline nitrogen, two deprotonated amide nitrogen, and two thioether sulfur bind copper(II) in *cis*, *trans*, and *cis* orientations. The geometric parameters are listed below in the supplementary materials. The angles at the metal center between *cis*-positioned donor pairs span the range 80.23 (7) - 105.76 (8)° and are close to those reported for related complexes (Singh & Mukherjee, 2005; Zhang *et al.*, 2004). The three *trans* angles, N1—Cu—N1^i^ 171.46 (10)°, N2—Cu—S 163.79 (5)°, and N2^i^—Cu—Si 163.79 (5)°, deviate significantly from the ideal value of 180° for a regular octahedral structure. This is presumably due to the structural demands imparted by the hexadentate ligand. The dimethylene bridge of the five-membered CuS_2_C_2_ ring has *gauche* conformation. The equatorial plane is occupied by two N atoms from quinoline moieties at longer Cu–N distances [2.183 (2) Å] and two thioether sulfur atoms [2.523 (1) Å]. The axial positions are occupied by the two amido nitrogen atoms at shorter Cu–N1 distances [1.956 (2) Å]. This Cu–N1 bond distance lies in the range of normal values for copper(II) to deprotonated amido nitrogen bond distances (Sunatsuki *et al.*, 1998). On the other hand, Cu–N2 bond distance is longer than normal value of 1.96–2.08 Å for the copper(II) to pyridyl nitrogen in related complexes. (Singh & Mukherjee, 2005; Sunatsuki *et al.*, 1998; Zhang *et al.*, 2004). In agreement with findings on a pair of analogous Cu and Ni complexes with pyridine replacing quinoline in the bqdapte ligand (Sunatsuki *et al.*, 1998), the coordination of the copper(II) ion in the title compound can be described as a Jahn-Teller distorted axially compressed (N1 and N1^i^) and equatorially elongated octahedron (N2, S, N2^i^, S^i^).

### Experimental

The ligand 1,4-bis[*o*-(quinoline-2-carboxamidophenyl)]-1,4-dithiobutane (H_2_bqctb) was prepared according to a general method reported elsewhere (Meghdadi *et al.*, 2011) by the reaction of quinaldic acid with 1,2-di(*o*-aminophenylthio)ethane (dapte) in the presence of triphenyl phosphite (TPP) and in tetrabutylammonium bromide (TBAB) as the reaction media.

The title complex was prepared as follows. To a stirring solution of H_2_bqctb (58.6 mg, 0.1 mmol) in dichloromethane (20 ml) was added a solution of Cu(CH_3_COO)_2_.H_2_O (20 mg, 0.1 mmol) in methanol (20 ml), and the mixture was stirred for 4 h. The final reaction mixture was filtered and the filtrate was left undisturbed for 24 h. Bright green crystals suitable for X-ray crystallography were obtained by slow evaporation of the filtrate at room temperature. The crystals were filtered off and washed with cold diethyl ether-dichloromethane (9/1), and dried under vacuum. Yield: 71%.

### Refinement

Refinement of *F*^2^ against ALL reflections. The weighted *R*-factor *wR* and goodness of fit *S* are based on *F*^2^, conventional *R*-factors *R* are based on *F*, with *F* set to zero for negative *F*^2^. The threshold expression of *F*^2^ > σ(*F*^2^) is used only for calculating *R* factors(gt) *etc*. and is not relevant to the choice of reflections for refinement. *R*-factors based on *F*^2^ are statistically about twice as large as those based on *F*, and *R*- factors based on ALL data will be even larger.

### Crystal data

**Table:**

[Cu(C_34_H_24_N_4_O_2_S_2_)]	*D*_x_ = 1.501 Mg m^−^^3^
*M**_r_* = 648.23	Mo *K*α radiation, λ = 0.71073 Å
Orthorhombic, *P**c**c**n*	Cell parameters from 118 reflections
*a* = 11.4124 (15) Å	θ = 17.8–27.3°
*b* = 13.5097 (18) Å	µ = 0.95 mm^−^^1^
*c* = 18.606 (2) Å	*T* = 150 K
*V* = 2868.6 (7) Å^3^	Plate, green
*Z* = 4	0.3 × 0.25 × 0.08 mm
*F*(000) = 1332	

### Data collection

**Table:**

Bruker SMART 100 diffractometer	2926 independent reflections
Radiation source: fine-focus sealed tube	2467 reflections with *I* > 2σ(*I*)
graphite	*R*_int_ = 0.040
ω scans	θ_max_ = 26.4°, θ_min_ = 2.2°
Absorption correction: multi-scan (*SADABS*; Bruker, 2003)	*h* = −14→14
*T*_min_ = 0.770, *T*_max_ = 0.927	*k* = −16→15
21464 measured reflections	*l* = −23→23

### Refinement

**Table:**

Refinement on *F*^2^	Primary atom site location: structure-invariant direct methods
Least-squares matrix: full	Secondary atom site location: difference Fourier map
*R*[*F*^2^ > 2σ(*F*^2^)] = 0.041	Hydrogen site location: inferred from neighbouring sites
*wR*(*F*^2^) = 0.119	H-atom parameters constrained
*S* = 1.22	*w* = 1/[σ^2^(*F*_o_^2^) + (0.072*P*)^2^] where *P* = (*F*_o_^2^ + 2*F*_c_^2^)/3
2926 reflections	(Δ/σ)_max_ = 0.001
195 parameters	Δρ_max_ = 0.83 e Å^−^^3^
0 restraints	Δρ_min_ = −0.40 e Å^−^^3^

### Special details

**Table:**

Geometry. All e.s.d.'s (except the e.s.d. in the dihedral angle between two l.s. planes) are estimated using the full covariance matrix. The cell e.s.d.'s are taken into account individually in the estimation of e.s.d.'s in distances, angles and torsion angles; correlations between e.s.d.'s in cell parameters are only used when they are defined by crystal symmetry. An approximate (isotropic) treatment of cell e.s.d.'s is used for estimating e.s.d.'s involving l.s. planes.
Refinement. Refinement of *F*^2^ against ALL reflections. The weighted *R*-factor *wR* and goodness of fit *S* are based on *F*^2^, conventional *R*-factors *R* are based on *F*, with *F* set to zero for negative *F*^2^. The threshold expression of *F*^2^ > σ(*F*^2^) is used only for calculating *R*-factors(gt) *etc*. and is not relevant to the choice of reflections for refinement. *R*-factors based on *F*^2^ are statistically about twice as large as those based on *F*, and *R*– factors based on ALL data will be even larger.

### Fractional atomic coordinates and isotropic or equivalent isotropic displacement parameters (Å^2^)

**Table:**

	*x*	*y*	*z*	*U*_iso_*/*U*_eq_	
C1	0.95359 (19)	0.63312 (18)	0.40463 (12)	0.0287 (5)	
C2	0.97185 (18)	0.72050 (18)	0.45446 (11)	0.0254 (5)	
C3	1.07936 (19)	0.72879 (19)	0.49149 (13)	0.0324 (6)	
H3	1.1395	0.6837	0.4832	0.039*	
C4	1.09359 (19)	0.80390 (19)	0.53956 (12)	0.0326 (6)	
H4	1.1642	0.8112	0.5639	0.039*	
C5	1.00127 (18)	0.87021 (17)	0.55221 (11)	0.0267 (5)	
C6	1.0075 (2)	0.9476 (2)	0.60300 (13)	0.0329 (6)	
H6	1.0766	0.9576	0.6285	0.040*	
C7	0.9143 (2)	1.0077 (2)	0.61517 (12)	0.0336 (6)	
H7	0.9200	1.0579	0.6492	0.040*	
C8	0.8095 (2)	0.99472 (18)	0.57677 (13)	0.0320 (5)	
H8	0.7460	1.0360	0.5858	0.038*	
C9	0.80010 (19)	0.92180 (18)	0.52621 (12)	0.0284 (5)	
H9	0.7308	0.9146	0.5004	0.034*	
C10	0.89525 (18)	0.85719 (17)	0.51284 (11)	0.0236 (5)	
C11	0.81073 (18)	0.54899 (17)	0.33282 (11)	0.0260 (5)	
C12	0.85951 (19)	0.45376 (19)	0.33824 (13)	0.0321 (5)	
H12	0.9200	0.4426	0.3707	0.039*	
C13	0.8195 (2)	0.37658 (19)	0.29635 (13)	0.0358 (6)	
H13	0.8535	0.3143	0.3010	0.043*	
C14	0.7295 (2)	0.39057 (19)	0.24748 (13)	0.0340 (6)	
H14	0.7045	0.3385	0.2186	0.041*	
C15	0.6777 (2)	0.48159 (18)	0.24211 (12)	0.0309 (5)	
H15	0.6162	0.4909	0.2100	0.037*	
C16	0.71616 (19)	0.56053 (18)	0.28432 (12)	0.0267 (5)	
C17	0.6849 (2)	0.7377 (2)	0.20459 (13)	0.0427 (7)	
H17A	0.6677	0.6982	0.1623	0.051*	
H17B	0.6410	0.7989	0.2005	0.051*	
Cu	0.7500	0.7500	0.38326 (2)	0.02369 (16)	
N1	0.84546 (16)	0.63022 (15)	0.37545 (9)	0.0242 (4)	
N2	0.88397 (15)	0.78305 (15)	0.46349 (9)	0.0245 (4)	
O	1.03533 (14)	0.57507 (15)	0.39668 (10)	0.0433 (5)	
S	0.63335 (5)	0.67137 (5)	0.28325 (3)	0.03301 (19)	

### Atomic displacement parameters (Å^2^)

**Table:**

	*U*^11^	*U*^22^	*U*^33^	*U*^12^	*U*^13^	*U*^23^
C1	0.0220 (11)	0.0413 (14)	0.0229 (11)	0.0030 (10)	0.0022 (9)	−0.0047 (10)
C2	0.0188 (10)	0.0374 (12)	0.0199 (10)	0.0012 (9)	−0.0004 (8)	−0.0011 (9)
C3	0.0200 (11)	0.0451 (14)	0.0321 (13)	0.0074 (10)	−0.0029 (9)	−0.0080 (11)
C4	0.0188 (11)	0.0490 (16)	0.0300 (12)	0.0028 (10)	−0.0053 (9)	−0.0047 (11)
C5	0.0225 (11)	0.0362 (13)	0.0212 (11)	0.0010 (9)	0.0002 (9)	0.0008 (9)
C6	0.0254 (12)	0.0450 (15)	0.0283 (12)	−0.0028 (10)	−0.0032 (9)	−0.0056 (11)
C7	0.0346 (13)	0.0385 (14)	0.0276 (12)	−0.0005 (11)	0.0019 (9)	−0.0086 (10)
C8	0.0284 (12)	0.0345 (13)	0.0331 (13)	0.0059 (10)	0.0062 (10)	−0.0002 (10)
C9	0.0228 (11)	0.0370 (14)	0.0255 (11)	0.0042 (10)	0.0026 (9)	0.0017 (10)
C10	0.0206 (10)	0.0320 (12)	0.0180 (10)	0.0008 (9)	0.0042 (8)	0.0027 (9)
C11	0.0222 (10)	0.0341 (13)	0.0216 (11)	−0.0017 (9)	0.0040 (9)	−0.0018 (9)
C12	0.0274 (11)	0.0410 (14)	0.0280 (12)	−0.0023 (10)	0.0020 (10)	0.0032 (11)
C13	0.0402 (13)	0.0314 (14)	0.0359 (13)	−0.0009 (11)	0.0079 (11)	0.0008 (10)
C14	0.0420 (14)	0.0330 (14)	0.0269 (12)	−0.0085 (11)	0.0052 (10)	−0.0045 (10)
C15	0.0327 (12)	0.0388 (14)	0.0212 (11)	−0.0093 (10)	0.0022 (9)	−0.0009 (10)
C16	0.0244 (10)	0.0352 (13)	0.0206 (11)	−0.0021 (9)	0.0028 (9)	−0.0006 (9)
C17	0.0633 (18)	0.0398 (16)	0.0249 (13)	0.0066 (13)	−0.0162 (12)	−0.0024 (10)
Cu	0.0172 (2)	0.0340 (3)	0.0199 (2)	−0.00048 (15)	0.000	0.000
N1	0.0192 (9)	0.0337 (11)	0.0199 (9)	0.0004 (8)	0.0008 (7)	−0.0029 (8)
N2	0.0174 (8)	0.0361 (10)	0.0201 (9)	0.0009 (8)	0.0012 (7)	0.0021 (8)
O	0.0219 (9)	0.0598 (13)	0.0481 (11)	0.0127 (8)	−0.0052 (8)	−0.0262 (9)
S	0.0244 (3)	0.0392 (4)	0.0354 (4)	0.0007 (2)	−0.0049 (2)	−0.0076 (3)

### Geometric parameters (Å, °)

**Table:**

C1—O	1.228 (3)	C11—N1	1.411 (3)
C1—N1	1.349 (3)	C11—C16	1.415 (3)
C1—C2	1.515 (3)	C12—C13	1.379 (4)
C2—N2	1.322 (3)	C12—H12	0.9300
C2—C3	1.412 (3)	C13—C14	1.385 (4)
C3—C4	1.362 (3)	C13—H13	0.9300
C3—H3	0.9300	C14—C15	1.368 (4)
C4—C5	1.403 (3)	C14—H14	0.9300
C4—H4	0.9300	C15—C16	1.395 (3)
C5—C6	1.411 (3)	C15—H15	0.9300
C5—C10	1.425 (3)	C16—S	1.771 (2)
C6—C7	1.357 (3)	C17—C17^i^	1.523 (6)
C6—H6	0.9300	C17—S	1.814 (3)
C7—C8	1.405 (3)	C17—H17A	0.9700
C7—H7	0.9300	C17—H17B	0.9700
C8—C9	1.366 (3)	Cu—N1^i^	1.9561 (19)
C8—H8	0.9300	Cu—N1	1.956 (2)
C9—C10	1.415 (3)	Cu—N2	2.1830 (18)
C9—H9	0.9300	Cu—N2^i^	2.1830 (18)
C10—N2	1.365 (3)	Cu—S	2.5225 (7)
C11—C12	1.405 (3)	Cu—S^i^	2.5225 (7)
			
O—C1—N1	128.9 (2)	C15—C14—C13	119.4 (2)
O—C1—C2	117.83 (19)	C15—C14—H14	120.3
N1—C1—C2	113.24 (19)	C13—C14—H14	120.3
N2—C2—C3	123.1 (2)	C14—C15—C16	120.6 (2)
N2—C2—C1	118.12 (19)	C14—C15—H15	119.7
C3—C2—C1	118.7 (2)	C16—C15—H15	119.7
C4—C3—C2	118.9 (2)	C15—C16—C11	121.0 (2)
C4—C3—H3	120.6	C15—C16—S	118.16 (17)
C2—C3—H3	120.6	C11—C16—S	120.48 (17)
C3—C4—C5	119.7 (2)	C17^i^—C17—S	115.10 (14)
C3—C4—H4	120.1	C17^i^—C17—H17A	108.5
C5—C4—H4	120.1	S—C17—H17A	108.5
C4—C5—C6	123.2 (2)	C17^i^—C17—H17B	108.5
C4—C5—C10	118.2 (2)	S—C17—H17B	108.5
C6—C5—C10	118.6 (2)	H17A—C17—H17B	107.5
C7—C6—C5	121.0 (2)	N1^i^—Cu—N1	171.48 (10)
C7—C6—H6	119.5	N1^i^—Cu—N2	105.76 (8)
C5—C6—H6	119.5	N1—Cu—N2	80.21 (7)
C6—C7—C8	120.5 (2)	N1^i^—Cu—N2^i^	80.21 (7)
C6—C7—H7	119.7	N1—Cu—N2^i^	105.76 (8)
C8—C7—H7	119.7	N2—Cu—N2^i^	93.71 (9)
C9—C8—C7	120.5 (2)	N1^i^—Cu—S	89.98 (6)
C9—C8—H8	119.8	N1—Cu—S	83.73 (5)
C7—C8—H8	119.8	N2—Cu—S	163.78 (5)
C8—C9—C10	120.4 (2)	N2^i^—Cu—S	92.79 (5)
C8—C9—H9	119.8	N1^i^—Cu—S^i^	83.73 (5)
C10—C9—H9	119.8	N1—Cu—S^i^	89.98 (6)
N2—C10—C9	119.90 (19)	N2—Cu—S^i^	92.79 (5)
N2—C10—C5	121.08 (19)	N2^i^—Cu—S^i^	163.78 (5)
C9—C10—C5	119.0 (2)	S—Cu—S^i^	84.93 (3)
C12—C11—N1	124.1 (2)	C1—N1—C11	120.39 (19)
C12—C11—C16	116.7 (2)	C1—N1—Cu	117.11 (16)
N1—C11—C16	119.1 (2)	C11—N1—Cu	121.92 (14)
C13—C12—C11	121.4 (2)	C2—N2—C10	118.88 (18)
C13—C12—H12	119.3	C2—N2—Cu	108.28 (15)
C11—C12—H12	119.3	C10—N2—Cu	132.55 (14)
C12—C13—C14	120.9 (2)	C16—S—C17	104.70 (12)
C12—C13—H13	119.6	C16—S—Cu	93.79 (7)
C14—C13—H13	119.6	C17—S—Cu	102.48 (9)
			
O—C1—C2—N2	−179.7 (2)	S—Cu—N1—C1	162.05 (16)
N1—C1—C2—N2	−0.6 (3)	S^i^—Cu—N1—C1	77.15 (15)
O—C1—C2—C3	−2.6 (3)	N2—Cu—N1—C11	173.02 (17)
N1—C1—C2—C3	176.5 (2)	N2^i^—Cu—N1—C11	81.90 (16)
N2—C2—C3—C4	0.8 (4)	S—Cu—N1—C11	−9.24 (15)
C1—C2—C3—C4	−176.1 (2)	S^i^—Cu—N1—C11	−94.14 (16)
C2—C3—C4—C5	1.0 (4)	C3—C2—N2—C10	−2.6 (3)
C3—C4—C5—C6	177.6 (2)	C1—C2—N2—C10	174.38 (19)
C3—C4—C5—C10	−1.0 (3)	C3—C2—N2—Cu	171.95 (19)
C4—C5—C6—C7	−177.6 (2)	C1—C2—N2—Cu	−11.1 (2)
C10—C5—C6—C7	1.0 (4)	C9—C10—N2—C2	−176.6 (2)
C5—C6—C7—C8	−0.6 (4)	C5—C10—N2—C2	2.5 (3)
C6—C7—C8—C9	−0.5 (4)	C9—C10—N2—Cu	10.4 (3)
C7—C8—C9—C10	1.2 (4)	C5—C10—N2—Cu	−170.41 (15)
C8—C9—C10—N2	178.4 (2)	N1^i^—Cu—N2—C2	−159.59 (15)
C8—C9—C10—C5	−0.8 (3)	N1—Cu—N2—C2	14.17 (15)
C4—C5—C10—N2	−0.8 (3)	N2^i^—Cu—N2—C2	119.54 (17)
C6—C5—C10—N2	−179.5 (2)	S—Cu—N2—C2	6.1 (3)
C4—C5—C10—C9	178.4 (2)	S^i^—Cu—N2—C2	−75.33 (15)
C6—C5—C10—C9	−0.3 (3)	N1^i^—Cu—N2—C10	13.9 (2)
N1—C11—C12—C13	178.2 (2)	N1—Cu—N2—C10	−172.3 (2)
C16—C11—C12—C13	2.2 (3)	N2^i^—Cu—N2—C10	−66.97 (17)
C11—C12—C13—C14	−0.1 (4)	S—Cu—N2—C10	179.59 (13)
C12—C13—C14—C15	−1.7 (4)	S^i^—Cu—N2—C10	98.16 (19)
C13—C14—C15—C16	1.2 (3)	C15—C16—S—C17	−83.65 (19)
C14—C15—C16—C11	1.0 (3)	C11—C16—S—C17	103.38 (19)
C14—C15—C16—S	−171.92 (18)	C15—C16—S—Cu	172.39 (17)
C12—C11—C16—C15	−2.7 (3)	C11—C16—S—Cu	−0.59 (18)
N1—C11—C16—C15	−178.84 (18)	C17^i^—C17—S—C16	−59.6 (3)
C12—C11—C16—S	170.09 (16)	C17^i^—C17—S—Cu	37.8 (3)
N1—C11—C16—S	−6.1 (3)	N1^i^—Cu—S—C16	178.82 (9)
O—C1—N1—C11	4.6 (4)	N1—Cu—S—C16	4.58 (9)
C2—C1—N1—C11	−174.42 (18)	N2—Cu—S—C16	12.6 (2)
O—C1—N1—Cu	−166.8 (2)	N2^i^—Cu—S—C16	−100.98 (9)
C2—C1—N1—Cu	14.1 (2)	S^i^—Cu—S—C16	95.11 (7)
C12—C11—N1—C1	24.9 (3)	N1^i^—Cu—S—C17	72.85 (11)
C16—C11—N1—C1	−159.3 (2)	N1—Cu—S—C17	−101.39 (11)
C12—C11—N1—Cu	−164.14 (16)	N2—Cu—S—C17	−93.4 (2)
C16—C11—N1—Cu	11.7 (3)	N2^i^—Cu—S—C17	153.05 (11)
N2—Cu—N1—C1	−15.69 (15)	S^i^—Cu—S—C17	−10.85 (9)
N2^i^—Cu—N1—C1	−106.81 (16)		

Symmetry codes: (i) −*x*+3/2, −*y*+3/2, *z*.
